# Exiting after Brexit: public perceptions of future European Union member state departures

**DOI:** 10.1080/01402382.2022.2164135

**Published:** 2023-01-23

**Authors:** Joseph Ganderson

**Affiliations:** European Institute, London School of Economics, London, UK

**Keywords:** Brexit, European Union, public opinion, Euroscepticism, motivated reasoning

## Abstract

Public opinion scholarship suggests that Europeans broadly interpret Brexit as a cautionary fable rather than an encouraging blueprint to follow. Yet, Brexit singularly demonstrates the possibility of European disintegration, and is but one of multiple recent crises that have brought the potential for member state departures into focus. Drawing on new survey data from 16 countries and using logistic regressions, this article charts Europeans’ perceptions of the likelihood future EU exits over the next decade. It finds evidence of asymmetric motivated reasoning: Euroscepticism and pro-Brexit views strongly associate with perceiving exits likely, while among Europhiles this association is only ameliorated, not reversed. This reveals two gaps with repercussions for understanding EU public opinion dynamics. First, between Eurosceptic policy elites’ softened policy stances on exit and their supporters’ steadfast sense that further departures remain likely. Second, between Europhiles’ scepticism of Brexit and a residual lack of confidence in EU cohesion.

Just days after the UK’s referendum on EU membership in June 2016, a triumphant Nigel Farage assured the European Parliament that ‘the UK won’t be the last country to leave the EU’ and claimed that ‘we offer a beacon of hope to other European countries’ (*New Europe* 2016). Indeed, the vote for Brexit was immediately greeted with enthusiasm by a host of Eurosceptic leaders. Prominent figures on the German and Danish radical right hailed Britain’s ‘courage’; while in France, the Netherlands, Slovakia and Greece nationalist parties pledged referendums on the same terms for their own countries (Braun *et al.* 2016; Hobolt and De Vries 2016). With the EU reeling from the effects of successive economic and humanitarian crises and disorientated by the referendum result, concern about a domino effect was palpable (Laffan 2019). But the several years of protracted exit negotiations that followed were accompanied by heightened polarisation over EU Remain-Leave orientations in the UK and a comparatively sanguine atmosphere on the membership question across the EU27 (Glencross 2019). The referendum slated by David Cameron to settle the UK’s longstanding ‘European Question’ appeared for a time to supercharge it, breeding acute polarisation at home that not only did not spread to the remaining 27 members, but perhaps even reinforced their cohesion by demonstrating the costs and complexities of leaving (Chopin and Lequesne 2021).

This is an assessment broadly shared by scholarly studies on the Brexit domino effect in public opinion and the post-referendum cohesion of the EU27. To date, Brexit appears not to have increased public appetite for further departures (De Vries 2017; Hobolt *et al.* 2022; Walter 2021a). In the UK’s wake, the same Eurosceptics that hailed the referendum result have apparently responded to Brexit by moderating their policy stances, taking exit referendums off the table and instead pursuing differentiated membership via opt-outs, brakes and selective ‘de-integration’ (Miró *et al.* 2023; van Kessel *et al.* 2020). Empirical indexing of present exits threats also indicates no state exhibiting anything close to the UK’s political characteristics over the past decade (Gastinger 2021).

This all suggests that British ‘awkwardness’ towards Europe might now be compartmentalised by EU leaders (George 1998). Yet equally, Brexit cannot be considered the only road to European disintegration, a process that might assume different forms from multiple origins (Alexander Shaw 2023; Leruth *et al.* 2019; Vollaard 2014; Webber 2019). The EU’s long decade of crisis, amidst which Brexit was affirmed by the British public, has repeatedly shown how ‘policy crises’ can escalate to become existential moments for the entire polity (Ferrera 2022). Faced with the mortality of sacred EU institutions or the potential for further member state exits during the Brexit, Euro area, migration and Covid-19 crises, European leaders have worked concertedly to hold the EU polity together (Ferrera 2022). But there is little certainty about whether these actions have convinced Europeans that another member exiting is as inconceivable to them as it was to EU leaders on the morning of 24^th^ June 2016. In short, the extent to which Brexit has effectively sealed off all future modes of membership departure is an open question, and one with essential implications for the EU’s future.

This article contributes to this debate by examining public perceptions of the likelihood of future exits over the next decade. Do European citizens believe that another member state might leave the EU in the coming years? Are these beliefs motivated by a normative desire to see this happen or a dispassionate or perhaps even rueful assessment that the EU27 is on a course towards further disintegration? And how closely are perceptions of future departures associated with assessments of the lived experience of Brexit’s impact on the UK? The article addresses these questions by drawing on new survey data on Brexit and the future of EU membership, offering insights from 15 diverse EU member states plus the UK.

Aggregate descriptive statistics reveal an overall tilt in public perceptions towards exit being more likely than not. Ordered logistic regressions conducted at the individual level show that underlying this is an ‘asymmetric’ pattern of motivated reasoning: both identifying Brexit positively, and especially with a general desire to leave to EU, is consistently associated with higher likelihoods of predicting another departure. Meanwhile, those with pro-European and anti-Brexit outlooks do not hold mirror opposite assessments. Their perception of future exits remains more likely than not, albeit not with the same confidence of Eurosceptics. Given the retreat of exit advocation by Eurosceptic elites and recent, broadly negative public perceptions of Brexit across the EU27 and increasingly so within the UK (Curtice 2021; *YouGov* 2022), this is surprising. After this lived experience of Brexit, one might have reasonably expected any asymmetric motivated reasoning to skew the opposite way, with triumphalist Europhiles and sober Eurosceptics projecting higher levels of member state cohesion and togetherness in the coming years.

Together, these results indicate that polarisation over the EU does not necessarily lead to predictably opposing patterns of empirical interpretation, and that motivated reasoning might be more present in one side than the other when making these assessments. These findings have implications both for scholarly understandings of European public opinion dynamics and for the EU proper. In a theory-refining sense, asymmetric motivated reasoning is hitherto absent from nascent studies of EU-Brexit public opinion, which typically identify a broadly bimodal distribution within populations between pro- and anti-EU opinion (Reinl and Evans 2021; Walter 2021b). These studies have focussed on normative opinions (‘how things should be’) rather than empirical assessments (‘how they will be’). By disaggregating the two, it is possible to tease out subtle but striking differences in the way Eurosceptics and Europhiles think and feel about the EU’s future.

The article’s findings could also have practical implications for the future of the Union. Expectations matter because they influence how those holding them behave, and this article’s results suggest that Europeans’ expectations might be formed in different ways depending on their priors. First, results suggest a misalignment between the post-Brexit moderation of Eurosceptic agendas and the steadfast aspiration-driven beliefs of their supporters. It hints that strong Eurosceptics might not have reconciled themselves to the inevitability of EU membership, even if strategic Eurosceptic policy elites have for the medium-term. As such, they are ripe for ‘reactivation’ should another circumstance prompt an entrepreneurial Eurosceptic call for exit in another member state. Second, it indicates that pro-Europeans and Brexit-sceptics retain a residual lack of confidence in the EU’s togetherness and possibly perceive further exits to be likely in spite of, not because of Brexit. This might suggest there are multiple sources of Europhile anxiety beyond Brexit, and a widespread perception that things could fall apart. Equally, it could be that Europhiles are sanguine about perceived departures of outlying or deviant states, departing from the geopolitical sensitivities of EU leaders concerned with both widening and deepening the Union to maximise their weight on the global stage.

The article proceeds as follows. It starts with a primer on the causes of member state exits and expert assessments of the presently low likelihood of this in the EU. It then juxtaposes two pertinent, contrasting logics of public belief formation – benchmarking and motivated reasoning – with close reference to recent scholarly contributions on EU public opinion on Brexit that have applied and synthesised them. Next, the article describes its data and presents preliminary national descriptive statistics before turning to individual predictors. Methodology is outlined and results of two models are described. The article concludes with a reflection on the implications of its findings for the EU’s current ‘polity-making’ agenda, and a call for further research.

## State of the art

### Disintegration, de-integration and the possibility of future exits

A starting point for any treatment of EU membership departures is the ‘supply-demand’ interplay between elites who reflect and form public opinion, and mass publics themselves. Examining how public opinion has increasingly challenged states’ membership of international organisations beyond Brexit, De Vries *et al.* (2021) suggest that latent public discontent must be activated by political entrepreneurs (typically ‘nationalist populists’) who are variously able to exploit national institutional opportunity structures (such as elections, referendums or party systems). Such entrepreneurialism may also be countervailed, as the multilateralism of the likes of Emmanuel Macron, Jacinda Ardern and Justin Trudeau has demonstrated (Hobolt *et al.* 2021). Yet, even if an underlying appetite or demand for revolt exists within a disgruntled or divided public, there is no guarantee that it will find the right elite, party system constellation to become a realistic prospect. Conversely, a cursory scan of EU history reveals that the existence of radical Euroscepticism among policy elites alone is no guarantor of disintegration. Even in the outlying ‘successful’ British case, there remains debate about the particularity, inevitability and contingency of the Brexit vote and its outcome (cf. Saunders 2016; Thompson 2017).

Yet, imitational neologisms (Italexit, Nexit, Frexit, Polexit and so on) have emerged in some countries after Brexit, suggesting further departures are conceivable. Much of this conjecture has been bound up in recent crisis events, through which EU leaders have worked to hold together the polity and forge compromises to smooth over threats of de-integration (‘spill-back’) or even potential wholesale exits (Ferrera 2022). During the Euro area crisis, Greece arrived at the brink of leaving the Euro, while the early months of Covid-19 saw a surge in Italian Euroscepticism that focussed the minds of European leaders (Truchlewski *et al.* 2021). Somewhat paradoxically, Brexit stands alone as the sole crisis that did culminate in an act of EU disintegration, but which might also be considered most affirming for the future of EU membership (Jones *et al.* 2021; Schelkle *et al.* 2023). This owes to detrimental political, economic and social fallout disproportionately hitting the departing state, prompting as noted the quiet abandonment of copycat calls for exit among Eurosceptic elites across the EU27 (Miró *et al.* 2023; van Kessel *et al.* 2020).

Exit potential is further complicated by governing elites in member states presently most consistently hostile to EU supranationalism – Poland and Hungary – purposefully not advocating departure, defying prevailing liberal democratic norms while retaining the trappings of membership (Closa 2023; Kelemen 2020). These leaders have started to actively depart from the EU’s characteristic technocratic depoliticisation, leaning into salient and polarising debates about the obligations of membership (Bressanelli *et al.* 2020). Most notably, in the wake of its 2021 law banning the ‘promotion or portrayal’ of homosexuality to under-18s, Dutch Prime Minister Mark Rutte said Hungary had ‘no business being in the EU anymore’ (*BBC News* 2021).

In short, the EU is now contending with two types of membership challenge that exhibit different dynamics. In some countries, it must manage discontent from nationalist populist oppositions that increasingly seek piecemeal ‘de-integration’. In others, it seeks to contain nationalist populist incumbents also intent on de-integration over exit, whose domestic policies have prompted outrage and attempts at externalisation. Both of these Eurosceptic dynamics signify threats to the future cohesion of the EU27 that exist quite apart from Brexit, but neither can be taken as simply indicative of any increased likelihood of further wholesale exits.

Overall, the abundant theorising that has emerged around disintegrative dynamics is not yet matched by empirical assessments of its actual or perceived likelihood, honourable exceptions aside. Hobolt (2016) analysed party systems and public opinion in five northern member states in the wake of the UK’s vote, judging them unlikely cases for emulation. Others have revisited public opinion using measures of UK Euroscepticism as a yardstick and found more mixed potential with substantial variance across states (Alexander Shaw 2023; Malloy *et al*. 2022). Gastinger’s (2021) ‘EU Exit Index’ represents the most comprehensive attempt to gauge the objective likelihood of further departures. It estimates how different underlying social sentiments, economic linkages and political institutions combine to determine different states’ exit prospects over time. Drawing on Eurobarometer indicators to construct an aggregate measure of exit likelihood across multiple relevant dimensions, it depicts the UK as consistently clear of all other member states after 2014. This holds across different weightings, while more likely cases (France, Czechia, Austria) require particular configurations of dynamic public opinion, economic and political institutions to move into a distant second place and have seen their own exit index scores decline through the 2010s. Gastinger (2021: 568) thus concludes that ‘the UK will likely remain the only country leaving the EU, at least in the foreseeable future’. How do ordinary Europeans feel about this question? Before sharing results, it is necessary to first introduce theories of public reasoning and what they predict.

### Brexit and EU27 public opinion: two logics of belief formation

Extensive literatures now exist on ongoing public support for and opposition to the EU and Brexit. The impact of Brexit on EU27 public opinion on the EU itself has, however, been a more niche scholarly concern. Within this, two leading theoretical lenses have emerged through which to interpret recent events: benchmarking and motivated reasoning. De Vries (2017) argues that a benchmarking theory of public opinion acutely applies to Brexit. She cites survey opinion and experimental data that suggest public support for the EU can ultimately be reduced to a cost-benefit calculation concerning EU membership versus a hypothetical alternate state of being outside. The UK after Brexit provides the first living example of such a state and thus valuable information to EU27 citizens seeking a tangible ‘benchmark’ against which to set their expectations of EU and state performance. De Vries (2017) finds that initial post-referendum uncertainty in British politics led to a small bounce in support among the EU27, but also that a more regular seesawing relationship should set in whereby Europeans losing faith in their own country’s institutions and government express increased support for Europe, and vice-versa. This, she suggests, underscores the importance of Eurosceptic entrepreneurs who ‘will be crucially important in framing what Brexit means in public debate’ (De Vries 2017: 25). To the extent that prominent Eurosceptic entrepreneurs in the Netherlands, Italy, France and elsewhere have all moderated their own calls for exit, this might be interpreted as a tacit acceptance that Brexit is not a blueprint, at least in the short-term, for the rest of the EU to follow (Chopin and Lequesne 2021).

The more recent work of Walter (2020, 2021a, 2021b) has offered the most sustained assessment of the impact of Brexit on EU public opinion, building on the results of a six-wave longitudinal survey of EU27 citizens conducted in six-monthly intervals between July 2017 and December 2019. Several findings emerge from this substantial dataset. The first concerns negotiating dynamics. EU citizens generally supported the hard stance taken by their governments during the Brexit negotiations, indeed they may even have motivated such a hard-line position (Chopin and Lequesne 2021). This ensured that attempts by the UK government to circumvent the European Commission and directly appeal to member states would be abortive from the start. However, this is caveated by instrumental and sentimental priors: those citizens most exposed to the fallout of Brexit (subjectively or objectively)^1^ favoured an accommodating stance; those most attached to the viability of the EU a harsh approach (Walter 2021a). When these perspectives collide, publics appeared well-aware of the tangible effects of Brexit and placed their own self-interest ahead of any ethereal attachment to the EU (Walter 2021a: 584).

Experimental studies have further isolated issue-based differences in benchmark weighting. For example, citizens are responsive to positive experimental cueing about potential sovereignty benefits deriving from Brexit, while being less moved by negative cueing about economic damage, with the overall effect being an increased optimism in citizens of other member states about a post-EU future (Hobolt *et al.* 2022). Complementing De Vries, these findings suggest that EU27 publics have exhibited a sensitivity to information about the effects of Brexit, sentimental and instrumental views on EU membership, and an apparently selective willingness to update their priors when cued with new information. Based on the findings described above that Brexit is perceived to be going badly for and inflicting net costs on the UK, and the fact that it has precipitated a moderation of elite Eurosceptic calls for exit, one can derive the following hypothesis for this article’s purposes:

H1 – Benchmarking: Negative evaluations of Brexit will be associated with lower future exit likelihood expectations, irrespective of views on the EU.

In essence, benchmarking ascribes to mass publics a rational, calculating if sometimes somewhat uneven responsiveness and willingness to update priors. The literature on motivated reasoning posits a contrast, instead emphasising how polarisation can radically distort the fundamental ways citizens update information and form opinions. Motivated reasoning ‘refers to the tendency to seek out information that confirms prior beliefs (i.e. a confirmation bias), view evidence consistent with prior opinions as stronger or more effective (i.e. a prior attitude effect), and spend more time arguing and dismissing evidence inconsistent with prior opinions, regardless of objective accuracy (i.e. a disconfirmation bias)’ (Druckman *et al.* 2013: 59). The implication being that in polarised populations, even well-informed citizens might be unevenly responsive to outcomes in disintegrative dynamics abroad, their interpretations of events inexorably filtered through their own desires (Bisgaard 2015).

Analysing views on the Brexit negotiations among people with Eurosceptic prior beliefs, Walter (2021a) finds that across the EU27 this demographic hoped the negotiations would deliver a clearer pathway for future exits, suggesting that EU leaders’ concerns about direct contagion effects are indeed well-founded. Subsequently, Walter (2021b) has engaged the contagion hypothesis more directly, testing it using EU27 and Swiss public opinion data. Results suggest that non-British voters are capable of being attentive to Brexit, updating their priors based on the ongoing reception of new information about how this process is unfolding. This is, however, moderated significantly by motivated reasoning among those at the margins on both sides. On Brexit, strong Eurosceptics and strong Europhiles each ‘react more strongly to developments that confirm their priors’ (Walter 2021b: 2398). This tallies with potentially underlying ‘affective polarisation’, the notion that polarised groups engage in stereotyping and out-grouping their political opponents, and are deeply invested in particular worldviews that are reinforced by the message of their own side’s policy elites (Gidron *et al.* 2020; Iyengar *et al.* 2019). Though identified closely with the contemporary United States, affective polarisation has also been located in post-referendum Britain (Hobolt *et al.* 2021), and to varying degrees across multiple EU member states (Reiljan 2020). Adapted to the current analysis, motivated reasoning should, then, associate exit likelihood with both Euroscepticism and pro-Brexit renderings.

H2 – Motivated Reasoning: Euroscepticism and positive evaluations of Brexit will be associated with higher future exit likelihood expectations.

### Further drivers of exit expectations

It should be noted that these primary hypotheses could be confounded by other stances. First, Europhiles might actively favour member state departures, privileging cohesion and the removal of outliers. This is a stance exemplified by Rutte’s rebuke of Orbán’s Hungary. Equally, as noted, criticism of the EU need not necessarily guide Eurosceptics to desire further departures, instead focussing their attention on returning powers to states and expanding the EU to include sympathetic new members such as Serbia, an intergovernmentalist model EU advocated by Orbán and published in European newspapers in June 2021 (*Hungary Today* 2021). This broaches the long-standing ‘wider versus deeper’ debate on EU development, modified for de-integration purposes to ‘narrower versus shallower’. Should the EU comprise a smaller number of more deeply integrated states, or a larger number of de-integrated states? This is an assessment of how respondents think the EU should look, rather than it will look, but the previous discussion clearly indicates that the former might motivate the latter.

H3 – Optimum Size: Preferences for a smaller EU will be associated with higher exit likelihood expectations, irrespective of views on Brexit and the EU.

Finally, a future membership crisis might be a function of some other exogenous or otherwise unforeseen shock, of the sort weathered by the EU over the past decade. Market, migration and pandemic crises have all seen the EU’s membership solidity seriously challenged in recent years (Ferrera 2022), and so ordinary Europeans might also harbour residual perceptions of EU fragility, irrespective of their other views on whether their own state should retain its membership.

H4 – Crisis Management: Negative perceptions of EU crisis response will be associated with higher exit likelihood expectation, irrespective of views on Brexit and the EU.

## Data and model

The article draws on a new survey, fielded between June and October 2021 to 2000 respondents in 15 EU member states of varying sizes and regions, plus the UK (total *N* = 32,000). Owing to its substantial length and concerns about overload, each respondent was randomly assigned two of five total modules relating to different crisis events and themes.^2^ After further pre-processing, including the removal of extremely rapid responses and those containing persistent ‘Don’t Know’ responses, this left 12,387 responses for the module related to Brexit and membership, split relatively evenly by country (see Table A1 – online appendix).

**Table 1.** Model results – log odds of ordinal logistic regressions.

	Exit likelihood	
	(1)	(2)
**Brexit: Bad for UK**		
Brexit: Good	0.778*** (0.065)	0.722*** (0.066)
Brexit: Neither/Nor	0.274*** (0.058)	0.267*** (0.059)
Brexit: Don’t Know	0.244*** (0.080)	0.209** (0.082)
**Education: Low**		
Education: Medium	−0.015 (0.082)	−0.066 (0.084)
Education: High	0.018 (0.083)	−0.067 (0.086)
**EU Referendum: Remain**		
EU Referendum: Leave	1.346*** (0.068)	1.194*** (0.072)
EU Referendum: Would Not Vote	0.524*** (0.094)	0.489*** (0.096)
EU Referendum: Don’t Know	0.514*** (0.086)	0.491*** (0.089)
**Transnationalism: Unconcerned**		
Transnationalism: Neutral	−0.172*** (0.057)	−0.151*** (0.058)
Transnationalism: Concerned	0.033 (0.052)	0.037 (0.053)
**Optimum Size: Smaller**		
Optimum Size: Right		−0.417*** (0.067)
Optimum Size: Larger		−0.463*** (0.076)
Optimum Size: Don’t Know		−0.360*** (0.083)
**Crisis Handling: Satisfied**		
Crisis Handling: Neutral		−0.061 (0.088)
Crisis Handling: Dissatisfied		0.169*** (0.049)
**Country: UK**		
Austria		−0.641*** (0.154)
Finland		−0.144 (0.159)
France		−0.779*** (0.155)
Germany		−0.526*** (0.159)
Greece		−0.298** (0.152)
Hungary		−0.557*** (0.150)
Ireland		−0.319** (0.154)
Italy		−0.481*** (0.158)
Latvia		−0.119 (0.157)
Netherlands		−0.299* (0.161)
Poland		−0.180 (0.156)
Portugal		−0.368** (0.152)
Romania		0.015 (0.154)
Spain		−0.682*** (0.154)
Sweden		−0.440*** (0.159)
Observations	8434	8434

Note: *p **p ***p < 0.01. Reference categories in bold.

### Descriptive results

**Figure 1.**
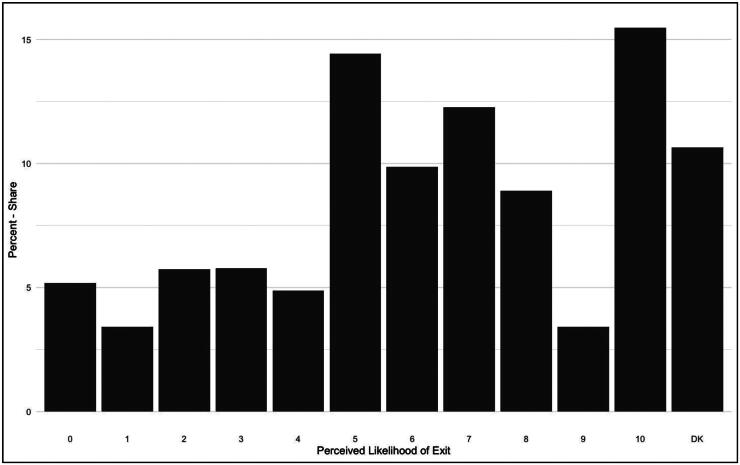
Relative frequencies: perceived likelihood of another EU Exit – next decade.

Figure 1 presents a preliminary assessment of how likely Europeans perceive another exit to be over the next decade.^3^ Contra expert assessments, Figure 1 shows slightly above medium levels of agreement with the notion that another member state will depart, with an unweighted mean of the 16 country means at 5.95. To be clear, such a value should not be overinterpreted as a strong indication of mass expectations of future exits, but it does at least demonstrate that respondents do not think of this outcome as unlikely. In all 16 states, a plurality of respondents agree that another departure is more likely than not. Mean scores vary from 5.2 in Spain to 6.7 in the UK. Within the EU, the highest mean score is in Latvia (6.4) closely followed by Finland and Romania (6.3). There is no notable bifurcation between extreme values across any countries (see Table A1 – online appendix for full country means and standard deviations). In sum, Figure 1 shows that Europeans have little confidence that another member will *not* depart in the next decade. Considering that only one member state has done so in its previous seven decades, that in review this is considered to have been a painful process borne out of unique features in UK politics, and that Brexit is thought to have moderated national populist calls for exit, this is a somewhat surprising initial finding.

**Figure 2.**
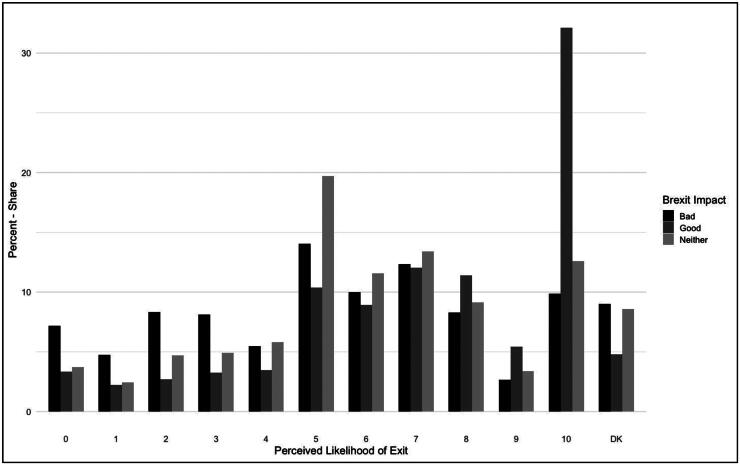
Relative frequencies, exit likelihood (by Brexit impact assessment).

Figure 2 offers an indication as to what is driving this higher likelihood distribution. Those believing Brexit to have been bad for the UK (51% of total respondents) show a fairly evenly distributed likelihood perception, and do not notably skew towards low scores, though here they do consistently outweigh those with a good (27%) or neutral neither/nor (22%) perception. The starkest group is the high share of pro-Brexit respondents signalling an extremely high perceived exit likelihood. This hints that perceiving Brexit negatively is largely decoupled from perceptions of further disintegration, but perceiving it positively is not. This association is explored in greater depth in the next section.

### Individual-level analysis

To complement these exploratory descriptive figures, the article now presents an individual level analysis. The dependent variable remains ‘likelihood of exit’. Though not phrased as a Likert form question, to simplify model results it can be repurposed broadly into three ordered categories: 0–4, *more unlikely than likely*; 5, *neither likely nor unlikely*; 6–10, *more likely than unlikely*. For ordinal dependent variables like this, ordered logistic regression is particularly appropriate (Long 1997). The analysis presents log odds of exit likelihood among individuals with positive perceptions of Brexit vis-à-vis those with negative views. Exponentiating these results, odds ratios and predicted probabilities show an individual’s likelihood of perceiving of future exit likelihoods at different levels given certain, potentially statistically significant, predictors (Brexit perception, Leave/Remain vote intention). However, this must first account for the wide array of confounding variables underlying both the dependent variable (Exit likelihood) and the key independent variable (Brexit perception).

**Figure 3.**
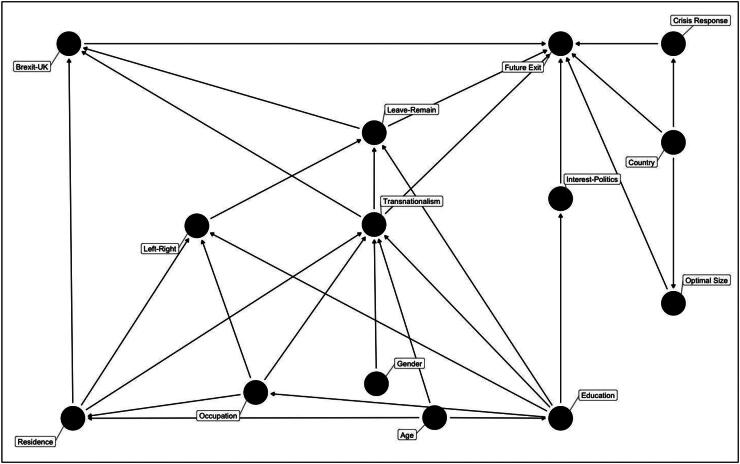
Directed acyclic graph – variable interaction assumptions.

In order to make sense of interactions between these variables, the article plots interactions using a graphical causal model that explicitly specifies and justifies its assumptions, then clarifies after adjustments which variables should be retained as controls in the final analysis. This avoids so-called ‘garbage can regression’ modelling (Achen 2005), which can lead to the inclusion of ‘bad controls’ that distort results, while also ensuring that essential ‘good controls’ are retained and omitted variable bias is avoided (Angrist and Pischke 2009; Cinelli *et al.* 2022). Figure 3 is a directed acyclic graph (DAG) specifying assumed links between the key independent, dependent and 12 further potential confounding variables across the country sample.

### Model assumptions

The DAG first specifies five socio-demographic characteristics that are said to underpin mid-level indicators of views, values and engagement in contemporary (EU) politics: residence (urban/rural), occupation, sex, age and education. Each of these variables correlate with views on transnationalism, or the GAL-TAN cleavage, while only some are now reliable indicators of conventional left-right ideological affinities (Marks *et al.* 2021). First, transnationalism and Euroscepticism is consistently manifest in variable geography, with more urban populations favouring European integration, economic openness, immigration and cultural plurality and those in smaller towns and rural communities being generally more hostile (Jennings and Stoker 2019; Maxwell 2019; Mitsch *et al.* 2021). Occupation, one historical proxy for class, is also said to inform one’s ideological outlook, propensity to vote for welfare maximising or tax reducing articles, and stance on transnationalism (Häusermann and Kriesi 2015; Kitschelt and Rehm 2014). Logically, it also goes some way to determining one’s options on the labour market and residence (Giannakis and Bruggeman 2020). In turn, education is consistently cited as a predictor of stances on Euroscepticism (Leave/Remain) and tolerance for transnationalism (Abou-Chadi and Hix 2021; Hakhverdian *et al.* 2013; van Elsas *et al.* 2016), though this association is not without its critics (cf. Kunst *et al.* 2020). However, rather than making a strong assumption of no link between education and Euroscepticism, left-right politics and transnationalism contra most conventional wisdom, for prudence they are assumed to be linked. Meanwhile, the influence of education on occupation is uncontroversial, and has been restated in a recent contribution by Iversen and Soskice (2019). Also comparatively uncontroversial is a hypothecated association between education and interest in politics (Le and Nguyen 2021), which in turn might influence the attention respondents pay to EU affairs and their motivated considerations of its membership fragility (Sarrasin *et al.* 2018).

While age and gender are now considered ‘inert’ on the left-right axis (Marks *et al.* 2021: 178), they do inform transnationalism. Younger Europeans are generally more likely to be highly educated than their parents (OECD 2015), and to gravitate towards urban locations (European Parliament 2010; Lee *et al.* 2018). Age and gender are also strongly associated with the transnational divide, with younger and female Europeans exhibiting generally higher levels of tolerance for transnationalism (Dolezal 2010).

There is assumed, then, to be a variable influence informing left-right and transnational political outlooks. Both of these mid-level variables in Figure 3 might be said to influence the key predictor (independent) and outcome (dependent) variables of interest. First, though the radical left has in recent years emerged in some quarters to challenge EU integration, left-right positioning remains a more reliable indicator of positions on Brexit and Euroscepticism (Van Elsas *et al.* 2016; van Elsas and van der Brug 2015; van Kessel *et al.* 2020) than it does of transnationalism more broadly, which comprises multiple cross-cutting dimensions that divide left from right (e.g. economic and cultural openness) (Hooghe and Marks 2018). Transnationalism is itself a strong predictor of one’s orientation towards the quintessential transnational project, the EU. This aligns it both to views on Brexit and referendum vote intention, and to perceptions of the likelihood of future exit as discussed above (De Vries 2017; Hobolt 2016; Hooghe and Marks 2018).

Finally, the neutral controls hypothesised about in the previous section include normative stance on the EU’s optimum size and evaluation of the EU’s response to era-defining crises. Though these are not likely to interact with perceptions of Brexit, they could be underpinned by national differences. Given clear country divides through the EU’s recent crises, it is reasonable to assume a link between country and crisis response (Hobolt and Wratil 2015; Taggart and Szczerbiak 2018), and there is evidence that populations at the country level have divergent views on EU expansion (Toshkov *et al.* 2014). All of the aforementioned model assumptions are collated with citations in Table A5 – online appendix.

Next, the R package ‘Dagitty’ (Textor *et al.* 2016) accounts for all hypothecated links and identifies a minimal sufficient adjustment set indicating that the variables *Education*, *Transnationalism* and *Leave-Remain* are potential confounders that must be controlled for.^4^ To these, the article adds the ‘neutral controls’ specified on the right of the DAG in a second model: optimal size, country, and an index of crisis response satisfaction. Assuming they do not interact with the main model, neutral controls may help further elucidate the analysis and open avenues for further research (Cinelli *et al.* 2022).

### Results

Individual level analysis comprises two ordinal logistic regressions, with raw log odd coefficients and standard errors summarised in Table 1. The first model shows strong and significant associations relating to individuals’ Brexit appraisal and referendum vote intention, but weak and non-significant results for education and a composite indicator of transnationalism. These findings hold when tested for multi-collinearity.^5^ A non-negative appraisal of Brexit’s impact on the UK, including uncertain (‘Don’t Know’ responses) is associated with an increased likelihood of expecting further exits over the next decade, with the strongest effect unsurprisingly found among those believing that Brexit has had a positive impact on the UK. For those holding this view, the log odds of perceiving further exits neutrally or likely is 0.78 points higher than those with a negative view. However, perhaps more surprisingly, the effect is even stronger in relative terms for those with a Leave rather than a Remain vote intention (1.35 points). Supporting H2, this suggests that Eurosceptics’ willingness to imagine the future through the lens of their own desires is a potentially even stronger motivator than positive renderings of the UK as a model case to follow.

Table 1 also indicates that the significance of these results hold in the expanded model 2, albeit with slightly moderated log odd coefficients. This model also includes the neutral controls ‘country’, ‘optimal size’ and ‘crisis response satisfaction’. It finds further significant associations with higher exit likelihood among those that normatively desire a smaller EU and among those dissatisfied with crisis response relative to those with opposite views (somewhat supporting H3 and H4). Between the 16 countries, there is wide variation between the size, confidence intervals and significance of the log odd effects, but almost all indicate a lower likelihood to perceive exit than the reference country, the UK. That UK respondents would perceive likelihood to be comparatively likely might not be surprising, given that their country has been exposed to the process and motivated British Eurosceptics might be especially eager to will wholesale disintegration into reality. However, this does indicate that, at least in the UK, Brexit is not widely perceived to be a strong cautionary, or deterrent fable. Despite the vicissitudes and heightened polarisation of post-referendum British politics, the UK public still perceives EU membership as particularly fragile. Re-running the full model without UK respondents also leads to essentially no noticeable changes in results, suggesting that the perceptions of British respondents are not especially distorting (see Table A7 – online appendix).

**Figure 4.**
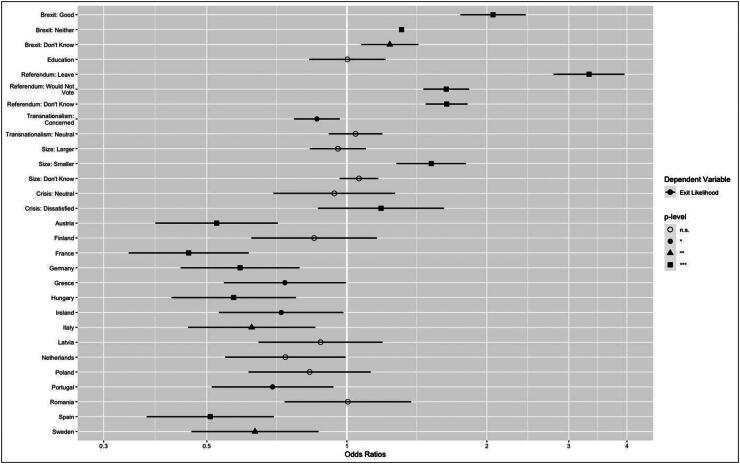
Odds ratios on exit likelihood – all variables and controls (Model 2).

**Figure 5.**
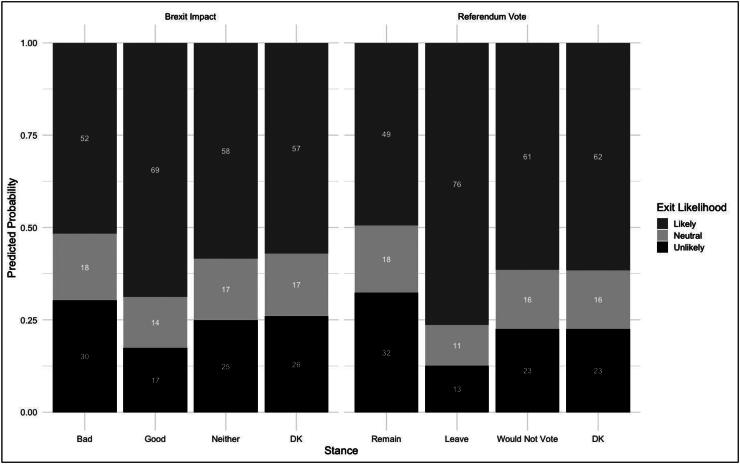
Predicted probability of exit likelihood perception by Brexit appraisal and referendum intention (Model 2).

These findings are more readily interpretable when exponentiated to produce odds ratios to demonstrate variance within independent variables and overall predicted probabilities of average opinions. These are displayed for the second model in Figures 4 and 5, and for the first in the online appendix. Figure 4 indicates that positive perceptions of Brexit and a desire to leave the EU increase the odds a respondent perceiving an exit as not unlikely by 2.1 and 3.3 times, respectively. These are the strongest significant effects, though holding an ambivalent view about Brexit (1.3 times), uncertainty (1.6) or abstinence concerning a membership referendum (1.6), a desire to see the EU shrink (1.5) and dissatisfaction with EU crisis handling (1.2) also increase exit perception likelihood relative to their respective reference positions. These lend some support to hypotheses H3 and H4, though the size of the effect appears moderate compared to core Brexit-Eurosceptic divides.

These skews, hypothesised in H2 and demonstrating a link between emotional and empirical assessments of the EU, are not entirely surprising, given the preceding discussion about how motivated reasoning may override more sober processing of political developments. The post-Brexit moderation of the radical right’s exit calls appears to hold little sway over their core demographic’s expectations. But two further reflections follow. First, separating Euroscepticism and Brexit assessments, normative views on the merits of membership outweigh perceptions of Brexit’s actual impact, somewhat undermining the benchmarking hypothesis (H1). This suggests that though Brexit might be seen as a model for departure by some Eurosceptics, it need not necessarily be the only path a departing state could follow. Indeed, those seeking to exit the EU might well still envision an alternative, potentially more successful, trajectory for their own country.

Second, these data only show the relative changes in positions by variable, but Figure 5 shows overall predicted probabilities of exit perception based on its two strongest predictors: Brexit and referendum intention. Here, an asymmetry is evident in the data: Brexit sceptical and Remain voters are still likely to perceive future exits. Based on the second, full model regression, there is only a 30% chance that a person with a negative Brexit perception sees further exits as unlikely, 52% likely; against 17% and 69%, respectively for those seeing Brexit as positive for the UK. The gap is wider for referendum intention: a 13% probability of a leave voter seeing another exit as unlikely, 32% for remain. Again, this illustrates a close coupling of normative and empirical calculations on the Eurosceptic side, but not among pro-Europeans, who might envisage further exits happening despite either potentially not wanting this outcome or being ambivalent or even positive about it as long as their own state’s membership is not in question.

## Conclusion

Recent scholarship on the contagion effects of Brexit on public opinion has quite comprehensively mapped how the UK’s vote has affected membership sentiments, bearing out some underlying theoretical explanations as to what informs voters’ decision making. While this trend might be subject to fluctuations as citizens update their information in the years to come, according primarily to present benchmarking assessments Brexit has acted more like a deterrent to exit than a model to follow. Yet, this has also been qualified by its cohabitation with motivated reasoning: those at the political margins tend to interpret Brexit and their own country’s European integration through the kaleidoscope of their desires, discounting contradictory and overweighting confirmatory evidence. This article drew on these recent theoretical and empirical insights on Brexit-EU public opinion and applied them using an empirical rather than a regular normative question on future membership departures over the next decade. Respondents in 16 European countries were asked: how likely is another member state departure over the next decade?

Results indicate that despite the historical idiosyncrasy of the UK’s membership, the domestically fraught nature of its exit process, and empirical analysis indicating the present unlikelihood of future departures, Europeans do see another exit as more likely than not. Literature on motivated reasoning in European integration and Brexit does not typically distinguish between two sides cleft by transnationalism, assuming predictable splits between pro- and anti-EU groups, but this article has suggested that attitudes may not always be mirror opposites. It identified an asymmetric pattern of motivated reasoning on this question between those expressing anti- and pro-European sentiments. Both positive feelings about Brexit’s impact on the UK and a leave vote intention are associated with higher expectations of a further departure, that the latter effect is even stronger suggests that those holding strong Eurosceptic desires also believe that these will come to fruition via a further form of member state disintegration. These effects are only moderated, but not reversed among pro-Europeans, those appraising Brexit negatively for the UK and wishing to remain. Overall, this group indicate a sentiment that even a badly perceived precedent in Brexit has not sealed off future departures among the EU27.

Evidently, further research is needed into the reasoning processes within these groups and the psycho-social origins of their differing expectations, and this article lacks the longitudinal panel data that would allow for a truly dynamic appraisal of Brexit’s impact on membership perceptions. Instead, it is intended as an initial enquiry, and further research tracking longitudinal shifts but also isolating whether individuals believe their own or another state will be likely to, or should leave, would be instructive. However, as an initial finding, the notion of asymmetric motivated reasoning offers hints at potentially meaningful implications for the EU, on both sides of the debate.

First, it suggests that those with anti-EU views may hold quite stridently to an expectation that their hopes will be realised, not adjusting to match the reality of a domestic party system where even radical Eurosceptics have largely reconciled themselves to working within the confines of the EU (van Kessel *et al.* 2020). Though most prominent national populist leaders around Europe are not presently advocating for exit referendums, a substantial public is apparently still hoping for them and remains ripe for reactivation. Further research might enquire as to where this disintegrative optimism is rooted: in insurgent domestic party systems, or a wider sense of EU dysfunction and contradiction (Jones *et al.* 2021)?

Perhaps more challenging is interpreting a relative lack of confidence in the EU27 holding together among Remainers and Brexit-sceptics. This group is less likely to perceive a future exit but still see this as more likely than not. Are everyday Europhiles simply more pessimistic about the future of the Union than experts, seeing multiple potential avenues to members departing? Confoundingly, Eurobarometer fieldwork conducted across the EU27 at the same time as this survey reported 12-year highs of optimism about the future of the EU (European Commission 2021). These data partially reflect a bounce from the challenges of Covid-19, but they may also indicate that in the minds of pro-Europeans there is no inherent contradiction between the positive future of the Union and the shedding of outlying members. After all, this status was once held by the UK, frustrating many Europhiles. If held, however, such a worldview would put everyday Europhiles at odds with EU leaders seeking to maximise the Union’s geographical scale and integrative depth, hence its geopolitical weight. Perhaps most concerning for these elites is a lack of concomitant motivated reasoning on their side of the argument. Despite widespread perceptions of Brexit as chastening for the UK, Europeans with quite different views on the EU itself appear quite unconvinced that other states have been persuaded not to follow suit.

## Supplementary Material

Supplemental Material

## Notes

1 ‘Objective’ exposure is measured in projected regional GDP loss from a hard Brexit scenario.

2 For further information, see Box 1 (online appendix).

3 ‘How likely do you think it is that another member state will leave the EU in the next decade? Select a value from 0 to 10, where 0 means “not at all likely” and 10 means “very likely”’. This survey question immediately followed a series of questions about Brexit. For all wordings, see Table A6 (online appendix).

4 On the methodology underlying this algorithmic process, see van der Zander *et al.* (2014).

5 A separate logistic regression test with dummy numeric variables indicates generally low variance inflation factors across the four independent ­variables used in both models, suggesting a low likelihood of multi-collinearity: Brexit Success: 1.39, Education: 1.02, Referendum Vote: 1.39, Transnationalism: 1.02. A Pearson correlation indicates only a moderate relationship (0.47) between referendum vote intention and Brexit appraisal, with all other paired (non-country) variables showing weak correlations (see Figure A3, online appendix).

## References

[CIT0001] Abou-Chadi, Tarik, and Simon Hix (2021). ‘Brahmin Left versus Merchant Right? Education, Class, Multiparty Competition, and Redistribution in Western Europe’, *The British Journal of Sociology*, 72:1, 79–92. 33629741 10.1111/1468-4446.12834

[CIT1001] Achen, Christopher H. (2005). ‘Let’s put Garbage-can Regressions and Garbage-can Probits where they belong’, *Conflict Management and Peace Science*, 22:4, 327–39.

[CIT2001] Alexander Shaw, Kate (2023). ‘Exit and Voice at the Cosmopolitan-Communitarian Cleavage: Challenges for European polity maintenance after Brexit’, *West European Politics*, forthcoming.

[CIT3001] Angrist, Joshua D., and Jórg-Steffen Pischke (2009). *Mostly Harmless Econometrics: An Empiricist’s Companion*. Princeton: Princeton University Press.

[CIT0003] BBC News (2021). ‘Dutch PM Rutte: No Place in EU for Hungary with Anti-LGBT Law’, 24 June, available at https://www.bbc.co.uk/news/world-europe-57596263 (accessed 2 March 2022).

[CIT0004] Bisgaard, Martin (2015). ‘Bias Will Find a Way: Economic Perceptions, Attributions of Blame, and Partisan-Motivated Reasoning during Crisis’, *The Journal of Politics*, 77:3, 849–60.

[CIT0005] Braun, Daniela, Swen Hutter, and Alena Kerscher (2016). ‘What Type of Europe? The Salience of Polity and Policy Issues in European Parliament Elections’, *European Union Politics*, 17:4, 570–92.

[CIT0006] Bressanelli, Edoardo, Christel Koop, and Christine Reh (2020). ‘EU Actors under Pressure: Politicisation and Depoliticisation as Strategic Responses’, *Journal of European Public Policy*, 27:3, 329–41.

[CIT0007] Chopin, Thierry, and Christian Lequesne (2021). ‘Disintegration Reversed: Brexit and the Cohesiveness of the EU27’, *West European Politics*, forthcoming.

[CIT0008] Cinelli, Carlos, Andrew Forney, and Judea Pearl (2022). ‘A Crash Course in Good and Bad Controls’, *Sociological Methods & Research*.

[CIT1008] Closa, Carlos (2023). ‘Worse than Brexit. Defiant non-compliance on rule of law and the erosion of supranationalism’, *West European Politics*, forthcoming.

[CIT0010] Curtice, John (2021). ‘Has Brexit Been a Success? The Public’s Perspective’, UK in a Changing Europe/NatCen Report, June 2021, available at https://whatukthinks.org/eu/wp-content/uploads/2021/06/WUKT-Brexit-Analysis-v4.pdf (accessed 10 October 2022).

[CIT0011] De Vries, Catherine E. (2017). ‘Benchmarking Brexit: How the British Decision to Leave Shapes EU Public Opinion’, *JCMS: Journal of Common Market Studies*, 55:1, 38–53.

[CIT0012] De Vries, Catherine E., Sara B. Hobolt, and Stefanie Walter (2021). ‘Politicizing International Cooperation: The Mass Public, Political Entrepreneurs, and Political Opportunity Structures’, *International Organization*, 75:2, 306–32.

[CIT0013] Dolezal, Martin (2010). ‘Exploring the Stabilization of a Political Force: The Social and Attitudinal Basis of Green Parties in the Age of Globalization’, *West European Politics*, 33:3, 534–52.

[CIT0014] Druckman, James N., Erik Peterson, and Rune Slothuus (2013). ‘How Elite Partisan Polarization Affects Public Opinion Formation’, *American Political Science Review*, 107:1, 57–79.

[CIT0015] European Commission (2021). ‘Eurobarometer: Optimism about the Future of the EU at Its Highest since 2009’, 10 September, available at https://ec.europa.eu/commission/presscorner/detail/en/ip_21_4610 (accessed 4 March 2022).

[CIT0016] European Parliament (2010). ‘How to Promote the Role of Youth in Rural Areas of Europe?’, November 2010, available at https://www.europarl.europa.eu/RegData/etudes/note/join/2010/438620/IPOL-AGRI_NT(2010)438620_EN.pdf (accessed 4 March 2022).

[CIT0017] Ferrera, Maurizio (2022). ‘The European Union and Cross-National Solidarity: Safeguarding “Togetherness” in Hard Times’, *Review of Social Economy*, 1–25.

[CIT0018] Gastinger, Markus (2021). ‘Introducing the EU Exit Index Measuring Each Member State’s Propensity to Leave the European Union’, *European Union Politics*, 22:3, 566–85.

[CIT0019] George, Stephen (1998). *An Awkward Partner: Britain in the European Community*. 3rd ed. Oxford: Oxford University Press.

[CIT0020] Giannakis, Elias, and Adriana Bruggeman (2020). ‘Regional Disparities in Economic Resilience in the European Union across the Urban–Rural Divide’, *Regional Studies*, 54:9, 1200–13.

[CIT0021] Gidron, Noam, James Adams, and Will Horne (2020). *American Affective Polarization in Comparative Perspective*. Cambridge: Cambridge University Press.

[CIT0022] Glencross, Andrew (2019). ‘The Impact of the Article 50 Talks on the EU: Risk Aversion and the Prospects for Further EU Disintegration’, *European View*, 18:2, 186–93.

[CIT0023] Hakhverdian, Armen, Erika van Elsas, Wouter van der Brug, and Theresa Kuhn (2013). ‘Euroscepticism and Education: A Longitudinal Study of 12 EU Member States, 1973–2010’, *European Union Politics*, 14:4, 522–41.

[CIT0024] Häusermann, Silja, and Hanspeter Kriesi (2015). ‘What Do Voters Want? Dimensions and Configurations in Individual-Level Preferences and Party Choice’, in Pablo Beramendi, Silja Hausermann, Herbert Kitschelt, and Hanspeter Kriesi (eds.), *The Politics of Advanced Capitalism*. Cambridge: Cambridge University Press, 202–30.

[CIT0025] Hobolt, Sara B. (2016). ‘The Brexit Vote: A Divided Nation, a Divided Continent’, *Journal of European Public Policy*, 23:9, 1259–77.

[CIT0026] Hobolt, Sara B., and Catherine E. de Vries (2016). ‘Public Support for European Integration’, *Annual Review of Political Science*, 19:1, 413–32.

[CIT0027] Hobolt, Sara B., Thomas J. Leeper, and James Tilley (2021). ‘Divided by the Vote: Affective Polarization in the Wake of the Brexit Referendum’, *British Journal of Political Science*, 51:4, 1476–93.

[CIT0028] Hobolt, Sara B., Sebastian A. Popa, Wouter van der Brug, and Hermann Schmitt (2022). ‘The Brexit Deterrent? How Member State Exit Shapes Public Support for the European Union’, *European Union Politics*, 23:1, 100–19.

[CIT0029] Hobolt, Sara B., and Christopher Wratil (2015). ‘Public Opinion and the Crisis: The Dynamics of Support for the Euro’, *Journal of European Public Policy*, 22:2, 238–56.

[CIT0031] Hooghe, Liesbet, and Gary Marks (2018). ‘Cleavage Theory Meets Europe’s Crises: Lipset, Rokkan, and the Transnational Cleavage’, *Journal of European Public Policy*, 25:1, 109–35.

[CIT0032] *Hungary Today* (2021). ‘Orbán Outlines His EU Vision in Full-Page Ads in Foreign Papers’, 28 June, available at https://hungarytoday.hu/orban-outlines-eu-vision-ads-foreign-papers/ (accessed 2 March 2022).

[CIT0033] Iversen, Torben, and David W. Soskice (2019). *Democracy and Prosperity: Reinventing Capitalism through a Turbulent Century*. Princeton: Princeton University Press.

[CIT0034] Iyengar, Shanto, Yphtach Lelkes, Matthew Levendusky, Neil Malhotra, and Sean J. Westwood (2019). ‘The Origins and Consequences of Affective Polarization in the United States’, *Annual Review of Political Science*, 22:1, 129–46.

[CIT0036] Jennings, Will, and Gerry Stoker (2019). ‘The Divergent Dynamics of Cities and Towns: Geographical Polarisation and Brexit’, *The Political Quarterly*, 90:S2, 155–66.

[CIT0037] Jones, Erik, R. Daniel Kelemen, and Sophie Meunier (2021). ‘Failing Forward? Crises and Patterns of European Integration’, *Journal of European Public Policy*, 28:10, 1519–36.

[CIT0039] Kelemen, R. Daniel (2020). ‘The European Union’s Authoritarian Equilibrium’, *Journal of European Public Policy*, 27:3, 481–99.

[CIT0041] Kitschelt, Herbert, and Philipp Rehm (2014). ‘Occupations as a Site of Political Preference Formation’, *Comparative Political Studies*, 47:12, 1670–706.

[CIT0043] Kunst, Sander, Theresa Kuhn, and Herman G. van de Werfhorst (2020). ‘Does Education Decrease Euroscepticism? A Regression Discontinuity Design Using Compulsory Schooling Reforms in Four European Countries’, *European Union Politics*, 21:1, 24–42.

[CIT0044] Laffan, Brigid (2019). ‘How the EU27 Came to Be’, *JCMS: Journal of Common Market Studies*, 57:1, 13–27.

[CIT1045] Le, Kien, and My Nguyen (2021). ‘Education and Political Engagemen’, *International Journal of Educational Development*, 85.

[CIT0045] Lee, Neil, Katy Morris, and Thomas Kemeny (2018). ‘Immobility and the Brexit Vote’, *Cambridge Journal of Regions, Economy and Society*, 11:1, 143–63.

[CIT0046] Leruth, Benjamin, Stefan Gänzle, and Jarle Trondal (2019). ‘Exploring Differentiated Disintegration in a Post-Brexit European Union’, *JCMS: Journal of Common Market Studies*, 57:5, 1013–30.

[CIT1048] Malloy, Brandon, Zeynep Ozkok, and Jonathan Rosborough (2022). ‘Is Brexit an outlier? Euroscepticism and public support for European integration’, *European Politics and Society*.

[CIT0048] Marks, Gary, David Attewell, Jan Rovny, and Liesbet Hooghe (2021). ‘Cleavage Theory’, in Marianne Riddervold, Jarle Trondal, and Akasemi Newsome (eds.), *The Palgrave Handbook of EU Crises*. New York: Springer International Publishing, 173–93.

[CIT0050] Maxwell, Rahsaan (2019). ‘Cosmopolitan Immigration Attitudes in Large European Cities: Contextual or Compositional Effects?’, *American Political Science Review*, 113:2, 456–74.

[CIT1053] Miró, Joan, Argyrios Altiparmakis, and Chendi Wang (2023). ‘The Short-Lived Hope for Contagion: Brexit in Social Media Communication of the Populist Right’, *West European Politics*, forthcoming.

[CIT0053] Mitsch, Frieder, Neil Lee, and Elizabeth Ralph-Morrow (2021). ‘Faith No More? The Divergence of Political Trust between Urban and Rural Europe’, *West European Politics*, 89, 102426.

[CIT0054] *New Europe* (2016). ‘Farage Wants a Sensible, Grown up Approach to Brexit Negotiations’, *New Europe*, Blog, 28 June, available https://www.neweurope.eu/article/farage-wants-a-sensible-grown-up-approach-to-brexit-negotiations/ (accessed 2 March 2022).

[CIT0055] OECD (2015). ‘Are Young People Attaining Higher Levels of Education than Their Parents?’, *Education Indicators in Focus*, 28.

[CIT0056] Reiljan, Andres (2020). ‘“Fear and Loathing across Party Lines” (Also) in Europe: Affective Polarisation in European Party Systems’, *European Journal of Political Research*, 59:2, 376–96.

[CIT0057] Reinl, Ann-Kathrin, and Geoffrey Evans (2021). ‘The Brexit Learning Effect? Brexit Negotiations and Attitudes towards Leaving the EU beyond the UK’, *Political Research Exchange*, 3:1.

[CIT0059] Sarrasin, Oriane, Theresa Kuhn, and Bram Lancee (2018). ‘What Explains Increasing Euroscepticism in Switzerland? A Longitudinal Analysis’, in R. Tillmann, M. Voorpostel, and P. Farago (eds.), *Social Dynamics in Swiss Society*. New York: Springer International Publishing, 203–14.

[CIT0060] Saunders, Robert (2016). ‘A Tale of Two Referendums: 1975 and 2016’, *The Political Quarterly*, 87:3, 318–22.

[CIT1060] Schelkle, Waltraud, Argyrios Altiparmakis, Joseph Ganderson, and Anna Kyriazi (2023). ‘Brexit – A Membership Crisis that Wasn’t’, *West European Politics*, forthcoming. 10.1080/01402382.2024.2325780 PMC11019451 38628814

[CIT0063] Taggart, Paul, and Aleks Szczerbiak (2018). ‘Putting Brexit into Perspective: The Effect of the Eurozone and Migration Crises and Brexit on Euroscepticism in European States’, *Journal of European Public Policy*, 25:8, 1194–214.

[CIT0064] Textor, Johannes, Benito van der Zander, Mark S. Gilthorpe, Maciej Liskiewicz, and George Th Ellison (2016). ‘Robust Causal Inference Using Directed Acyclic Graphs: The R Package “Dagitty”’, *International Journal of Epidemiology*, 45:6, 1887–94. 28089956 10.1093/ije/dyw341

[CIT0065] Thompson, Helen (2017). ‘Inevitability and Contingency: The Political Economy of Brexit’, *The British Journal of Politics and International Relations*, 19:3, 434–49.

[CIT0066] Toshkov, Dimiter, Elitsa Kortenska, Antoaneta Dimitrova, and Adam Fagan (2014). ‘The “Old” and the “New” Europeans: Analyses of Public Opinion on EU Enlargement in Review’, MAXCAP Working Paper, April 2014, available at https://userpage.fu-berlin.de/kfgeu/maxcap/system/files/maxcap_wp_02.pdf (accessed 10 October 2022).

[CIT0067] Truchlewski, Zbigniew, Waltraud Schelkle, and Joseph Ganderson (2021). ‘Buying Time for Democracies? European Union Emergency Politics in the Time of COVID-19’, *West European Politics*, 44:5-6, 1353–75.

[CIT0068] Van der Zander, Benito, Johannes Textor, and Maciej Liśkiewicz (2014). ‘Constructing Separators and Adjustment Sets in Ancestral Graphs’, *AUAI Press Working Paper*, available at https://auai.org/uai2014/proceedings/individuals/209.pdf (accessed 10 October 2022).

[CIT1069] Van Elsas, Erika, and Wouter van der Brug (2015). ‘The Changing Relationship between Left-Right Ideology and 3uroscepticism, 1973-2010’, *European Union Politics*, 16:2, 194–215.

[CIT0069] Van Elsas, Erika J., Armen Hakhverdian, and Wouter van der Brug (2016). ‘United against a Common Foe? The Nature and Origins of Euroscepticism among Left-Wing and Right-Wing Citizens’, *West European Politics*, 39:6, 1181–204.

[CIT0070] Van Kessel, Stijn, Nicola Chelotti, Helen Drake, Juan Roch, and Patricia Rodi (2020). ‘Eager to Leave? Populist Radical Right Parties’ Responses to the UK’s Brexit Vote’, *The British Journal of Politics and International Relations*, 22:1, 65–84.

[CIT0071] Vollaard, Hans (2014). ‘Explaining European Disintegration’, *JCMS: Journal of Common Market Studies*, 52:5, 1142–59.

[CIT0072] Walter, Stefanie (2020). ‘The Mass Politics of International Disintegration’, CIS Working Paper, 105, available at https://ethz.ch/content/dam/ethz/special-interest/gess/cis/cis-dam/CIS_2020/WP%20105%20Walter.pdf (accessed 11 January 2022).

[CIT0073] Walter, Stefanie (2021a). ‘EU-27 Public Opinion on Brexit’, *JCMS: Journal of Common Market Studies*, 59:3, 569–88. 10.1111/jcms.13107 PMC9292232 35875409

[CIT0074] Walter, Stefanie (2021b). ‘Brexit Domino? The Political Contagion Effects of Voter-Endorsed Withdrawals from International Institutions’, *Comparative Political Studies*, 54:13, 2382–415.

[CIT0075] Webber, Douglas (2019). *European Disintegration? The Politics of Crisis in the European Union*. London: Red Globe Press.

[CIT0076] *YouGov* (2022). ‘One in Five Who Voted for Brexit Now Think It Was the Wrong Decision’, November 17, Blog, available at https://yougov.co.uk/topics/politics/articles-reports/2022/11/17/one-five-who-voted-brexit-now-think-it-was-wrong-d (accessed 10 October 2022).

