# Supplementary material for: Exiting after Brexit: public perceptions of future European Union member state departures
Source: West Eur Polit. 2023 Jan 23;47(5):1199–222. doi: 10.1080/01402382.2022.2164135 (PMC11018069; doi:10.1080/01402382.2022.2164135)
Supplement: Supplemental Material [file FWEP_A_2164135_SM9122.pdf]

Exiting after Brexit: Public Perceptions of Future European Union Member State Departures

Joseph Ganderson, *European Institute, London School of Economics, UK*

Online Appendix

Box 1 – Survey Information

The survey data used in this paper were drawn from part of a more extensive survey on recent EU crisis politics, covering 16 European countries. Fieldwork was conducted mostly in June 2021, with trials and quota balancing supplementary samples conducted in May and October 2021 respectively (see Table A1). These countries were selected primarily for their exposure to or direct involvement in one or more major EU crisis events over the past decade, but also to ensure regional and size-based variation. Owing to budgetary and sampling limitations, unfortunately it was not possible to survey all 28 states. The lengthy and demanding nature of the survey meant that each respondent only answered two of five in-depth modules, including the Brexit-Membership Crisis module containing the outcome (Exit Likelihood) and predictor (Brexit Success) questions.

- **Vendor:** Gallup Europe
- **Countries (Available Languages):**
  - Austria (German)
  - Finland (Finnish, Swedish)
  - France (French)
  - Germany (German)
  - Greece (Greek)
  - Hungary (Hungarian)
  - Ireland (English)
  - Italy (Italian)
  - Latvia (Latvian, Russian)
  - Netherlands (Dutch)
  - Poland (Polish)
  - Portugal (Portuguese)
  - Romania (Romanian)
  - Spain (Spanish)
  - Sweden (Swedish)
  - UK (English)
- **Fieldwork:** 16-29/30 June 2021 in all countries unless stated (see Table A1)
- **Data Collection:** CAWI (Online only)
- **Respondents Per Country:** 2000 (32,000 Total)
- **Weighting Variables:** Gender/Age/Education/Rural-Urban Location
- **Attention Checks:** No
- **Median Response Time (rounded minutes):** 21
- **Question Modules (All Respondents):**
  - Socio-Demographics
  - Political Attitudes
  - EU Crisis Attitudes (Comparative)
- **In-Depth Crisis Modules (40% of respondents per module, random ordering):**
  - ***Membership Crisis***
  - Covid Crisis
  - Social Crisis
  - Migration Crisis
  - Eurozone Crisis
- **Quota-Modulation:** Each respondent receives 2 of 5 in-depth crisis modules, ordered randomly

Table A1: Survey and Dependent Variable – Descriptive Statistics by Country

Country	N	Supplementary Fieldwork (N)	Median Response (Mins.)	Dependent Variable (Exit Likelihood)				
				Mean	Mode	St. Dev.	Don't Knows	Don’t Know (%)
Austria	787		21	5.91	10	2.96	53	6.73
Finland	764		20	6.39	10	2.88	48	6.28
France	817	5-16 Oct (56)	19	5.74	5	2.85	139	17.01
Germany	683		20	5.63	5	2.83	114	16.69
Greece	920	5-15 Oct (149)	22	6.26	10	2.88	71	7.72
Hungary	821	5-18 Oct (41)	24	5.56	5	2.98	72	8.77
Ireland	792	24-25 May (22)	21	5.63	10	3.11	83	10.48
Italy	747		19	6.01	10	2.86	86	11.51
Latvia	736		25	6.38	10	2.76	61	8.29
Netherlands	686		20	5.88	7	2.57	91	13.27
Poland	903	5-16 Oct (144)	21	5.91	5	2.98	106	11.74
Portugal	862	5-18 Oct (73)	23	5.33	5	2.99	100	11.61
Romania	850		22	6.30	10	3.09	62	7.29
Spain	789	5-19 Oct (88)	21	5.11	5	3.02	97	13.82
Sweden	714		21	6.10	10	2.93	81	11.34
UK	516		16	7.01	10	2.81	61	11.82
**Total**	**12387**			**5.95*<br>5.93**	**10**	**2.95**	**1325**	**10.77**

*Unweighted mean of country means. Otherwise, all total columns weighted by all data.

Table A2: Outcome Variable – Response Share by Country (Raw)

	Less Likely				< Exit Likelihood >			More Likely				
	0	1	2	3	4	5	6	7	8	9	10	DK
**Austria**	5.0	3.0	6.0	7.5	6.1	15.5	8.6	10.9	9.5	4.1	17.0	6.7
**Finland**	3.8	3.1	5.0	5.5	5.1	9.2	11.6	14.4	10.7	6.2	19.1	6.3
**France**	4.9	2.4	6.4	4.9	4.2	15.2	10.0	13.0	7.7	1.6	12.7	17.0
**Germany**	5.4	2.2	6.4	6.1	3.5	15.8	9.4	12.7	8.3	2.9	10.4	16.7
**Greece**	3.7	2.4	6.5	3.7	5.2	15.8	9.7	12.9	8.5	3.7	20.2	7.7
**Hungary**	6.0	5.4	6.1	6.0	4.3	18.6	9.3	10.5	8.5	2.8	13.9	8.8
**Ireland**	7.3	3.7	6.3	7.8	5.1	11.7	9.2	11.0	9.2	2.7	15.5	10.5
**Italy**	4.1	2.9	5.5	5.9	4.8	13.1	11.1	13.5	9.0	2.7	15.8	11.5
**Latvia**	1.8	3.3	5.0	5.0	4.3	17.5	7.9	14.3	8.6	4.3	19.7	8.3
**Netherlands**	3.4	1.9	5.8	6.0	5.4	12.0	11.4	18.2	11.4	3.2	8.2	13.3
**Poland**	4.4	5.0	5.4	5.2	3.4	16.1	9.7	10.0	8.7	4.7	15.6	11.7
**Portugal**	8.1	4.5	7.1	4.4	4.9	14.8	11.4	11.4	8.8	2.4	10.6	11.6
**Romania**	6.1	2.9	4.0	4.7	4.6	14.9	9.2	10.1	8.9	3.5	23.6	7.3
**Spain**	9.0	4.8	5.8	7.4	7.0	13.4	9.9	10.4	7.5	2.5	10.0	12.3
**Sweden**	4.1	3.1	4.5	7.6	5.5	11.6	9.8	12.5	8.4	3.9	17.8	11.3
**UK**	2.3	2.9	3.7	2.7	4.7	7.4	6.8	14.1	13.0	5.6	25.0	11.8
**Mean***	5.0	3.4	5.6	5.6	4.9	13.9	9.7	12.5	9.2	3.6	15.9	10.8

Table A3: Outcome Variable – Response Share by Country (Pooled)

	Less Likely (0-4)	Neutral (5)	More Likely (6-10)	DK
**Austria**	27.6	15.5	50.2	6.7
**Finland**	22.5	9.2	62.0	6.3
**France**	22.8	15.2	45.0	17.0
**Germany**	23.7	15.8	43.8	16.7
**Greece**	21.5	15.8	55.0	7.7
**Hungary**	27.6	18.6	44.9	8.8
**Ireland**	30.2	11.7	47.6	10.5
**Italy**	23.3	13.1	52.1	11.5
**Latvia**	19.4	17.5	54.8	8.3
**Netherlands**	22.4	12.0	52.3	13.3
**Poland**	23.5	16.1	48.7	11.7
**Portugal**	29.0	14.8	44.5	11.6
**Romania**	22.4	14.9	55.4	7.3
**Spain**	34.0	13.4	40.3	12.3
**Sweden**	24.6	11.6	52.4	11.3
**UK**	16.3	7.4	64.5	11.8
**Mean***	24.4	13.9	50.9	10.8

**Unweighted*

Table A4: Predictor and Confounders Variable – Response Total and Share

**Brexit Impact**	**Total**	**Share**
Bad	5555	44.9
Good	2898	23.4
Neither/Nor	2472	20
Don't Know	1462	11.8
**Education**		
Low	862	7
Medium	4444	35.9
High	4085	33
NA	2996	24.2
**EU Referendum**		
Remain	7854	63.4
Leave	2636	21.2
Would Not Vote	821	6.6
Don't Know	1076	8.7
**Transnationalism Index**		
Unconcerned	5505	44.4
Neutral	2672	21.6
Concerned	4210	34
**Optimum Size**		
Smaller	2352	19
About Right	5092	41.1
Larger	2757	22.3
Don't Know	2186	17.6
**Crisis Handling Index**		
Satisfied	5151	41.6
Neutral	855	6.9
Dissatisfied	6381	51.5

Table A5: DAG Model Assumptions (Article - Figure 3)

Connection	Citation
Brexit-UK -> Future Exit<br>Crisis Response -> Future Exit<br>Transnationalism -> Future Exit<br>Leave-Remain -> Future Exit<br>Country -> Future Exit<br>Optimal Size -> Future Exit<br>Interest-Politics -> Future Exit	Novel dependent variable, see paper.
Residence -> Brexit-UK<br>Residence -> Left-Right<br>Residence -> Transnationalism	Jennings and Stoker (2019), Maxwell (2019), Maxwell, R. (2019)<br>Mitsch et al (2021)
Occupation -> Left-Right	Kitschelt and Rehm (2014)
Occupation -> Residence	Giannakis and Bruggeman (2020)
Occupation -> Transnationalism	Häusermann, S., & Kriesi, H. (2015)
Education -> Leave-Remain	Cf. Hakhverdian et al (2012), Kunst et al (2019)
Education -> Left-Right	Hix and Abou Chadi (2021)
Education -> Transnationalism	Hakhverdian, A., van Elsas, E., van der Brug, W., & Kuhn, T. (2013).
Education -> Occupation	Iversen and Soskice (2020)
Education -> Interest-Politics	Le and Nguyen (2021)
Age -> Education	OECD (2015)
Age -> Residence	EU Parliament (2010), Lee et al (2018)
Age -> Transnationalism	Dolezal (2010), Marks et al (2019)
Gender -> Transnationalism	Dolezal (2010), Marks et al (2021)
Left-Right -> Brexit-UK	Hakhverdian et al (2016)<br>van Elsas and van der Brug (2015)<br>van Kessel et al (2020)
Left-Right -> Leave-Remain	van Elsas and van der Brug (2015)
Leave-Remain -> Brexit-UK	Walter (2021)
Transnationalism -> Brexit-UK	Hooghe and Marks (2017), DeVries (2017), Hobolt (2016), Walter (2017)
Transnationalism -> Leave-Remain	Hooghe and Marks (2017), De Vries (2018)
Country -> Crisis Response	Hobolt and Wratil (2015), Taggart and Szczerbiak (2018)
Country -> Optimal Size	Toshkov et al (2014)

Table A6: Variables for Analysis

Variable		Survey Question(s)	Survey Response Scale	Analysis Categories (* OLR Reference)
**Outcome**	**Exit Likelihood**	How likely do you think it is that another member state will leave the EU in the next decade?	Not at all likely (0) – Very likely (10)	0-4: Unlikely<br>5: Neutral<br>6-10: Likely
**Predictor**	**Brexit Success**	Thinking about the UK's exit from the EU, known as 'Brexit', do you think this has been a good thing or a bad thing overall for the UK?	Bad*<br>Neither Good nor Bad<br>Good	
**Confounders**	**Education**	How old were you when you stopped full-time education?	Age (Years)	<=15: Low*<br>>15 & <=19: Medium<br>>19 High
	**Leave-Remain**	EU15: If a referendum on the continuation of [COUNTRY]'s EU membership were to be held today, how would you vote?<br>UK: Imagine the UK was still a member of the EU. If a referendum on the continuation of the UK's EU membership were to be held today, how would you vote?	Remain*<br>Leave<br>Would Not Vote	
	**Transnationalism**	Some people have concerns about the process of European integration. Using the below scale where "0" means you are very much concerned and "10" means that you are not afraid at all, how much you are currently afraid that European integration may cause:<br><br>**Economic:** The loss of jobs and social security in (COUNTRY)<br>**Cultural:** The loss of national identity and culture	Very much concerned (0) – Not at all concerned (10)	Index represented by mean value of economic and cultural dimensions. If only one value specified, this value is taken.<br><br>Concerned (mean <5)*<br>Neutral (mean = 5)<br>Unconcerned (mean > 5).
**Neutral Controls**	**Optimal Size**	Thinking about the 27 countries that are currently EU members, which of the following statements comes closest to your view:	There are too many countries in the EU and it should be smaller,<br>The number of countries in the EU is about right at the moment,<br>The EU should expand to take in more countries	
	**Country**		[Country location of respondent –	UK*

		auto-coded]	[All other countries]
**Crisis Response**	Generally speaking, how satisfied are you with the way the European Union has handled each of the following challenges?<br><br>**Euro:** Financial and economic issues in the years 2010-2012<br>**Migration:** Refugee flows in the years 2015-2016<br>**Social:** Poverty and unemployment in the decade 2010-2019<br>**Covid:** The Covid-19 Pandemic	Completely dissatisfied (0) – Completely satisfied (10)	Index represented by mean value of crises. If some values missing, mean of present values taken.<br><br>Unsatisfied (mean <5)*<br>Neutral (mean = 5)<br>Satisfied (mean > 5)

Table A7: Log Odds of Ordinal Logistic Regressions (No UK)

	Exit Likelihood	
	(1)	(2)
**Brexit: Bad for UK**		
Brexit: Good	0.751^{***} (0.066)	0.706^{***} (0.067)
Brexit: Neither/Nor	0.259^{***} (0.059)	0.250^{***} (0.060)
Brexit: Don't Know	0.257^{***} (0.080)	0.218^{***} (0.083)
**Education: Low**		
Education: Medium	-0.027 (0.084)	-0.091 (0.086)
Education: High	0.024 (0.085)	-0.075 (0.087)
**EU Referendum: Remain**		
EU Referendum: Leave	1.323^{***} (0.069)	1.199^{***} (0.073)
EU Referendum: Would Not Vote	0.537^{***} (0.096)	0.505^{***} (0.098)
EU Referendum: Don't Know	0.495^{***} (0.087)	0.478^{***} (0.090)
**Transnationalism: Unconcerned**		
Transnationalism: Neutral	-0.148^{**} (0.058)	-0.133^{**} (0.058)
Transnationalism: Concerned	0.064 (0.052)	0.063 (0.054)
**Optimum Size: Smaller**		
Optimum Size: Right		-0.371^{***} (0.068)
Optimum Size: Larger		-0.408^{***} (0.077)
Optimum Size: Don't Know		-0.344^{***} (0.085)
**Crisis Handling: Satisfied**		
Crisis Handling: Neutral		-0.054 (0.089)
Crisis Handling: Dissatisfied		0.174^{***} (0.049)
**Country: Ireland**		
Austria		-0.314^{**} (0.122)
Finland		0.175 (0.127)
France		-0.448^{***} (0.124)
Germany		-0.201 (0.128)
Greece		0.021 (0.120)
Hungary		-0.243^{**} (0.117)
Italy		-0.154 (0.128)
Latvia		0.199 (0.125)
Netherlands		0.034 (0.130)
Poland		0.134 (0.124)
Portugal		-0.060 (0.118)
Romania		0.327^{***} (0.121)
Spain		-0.369^{***} (0.121)
Sweden		-0.117 (0.129)
Observations	8,076	8,076

Note: *p**p***p<0.01. Reference categories in **bold**.

Figure A1: Odds Ratios for Full Model (1)

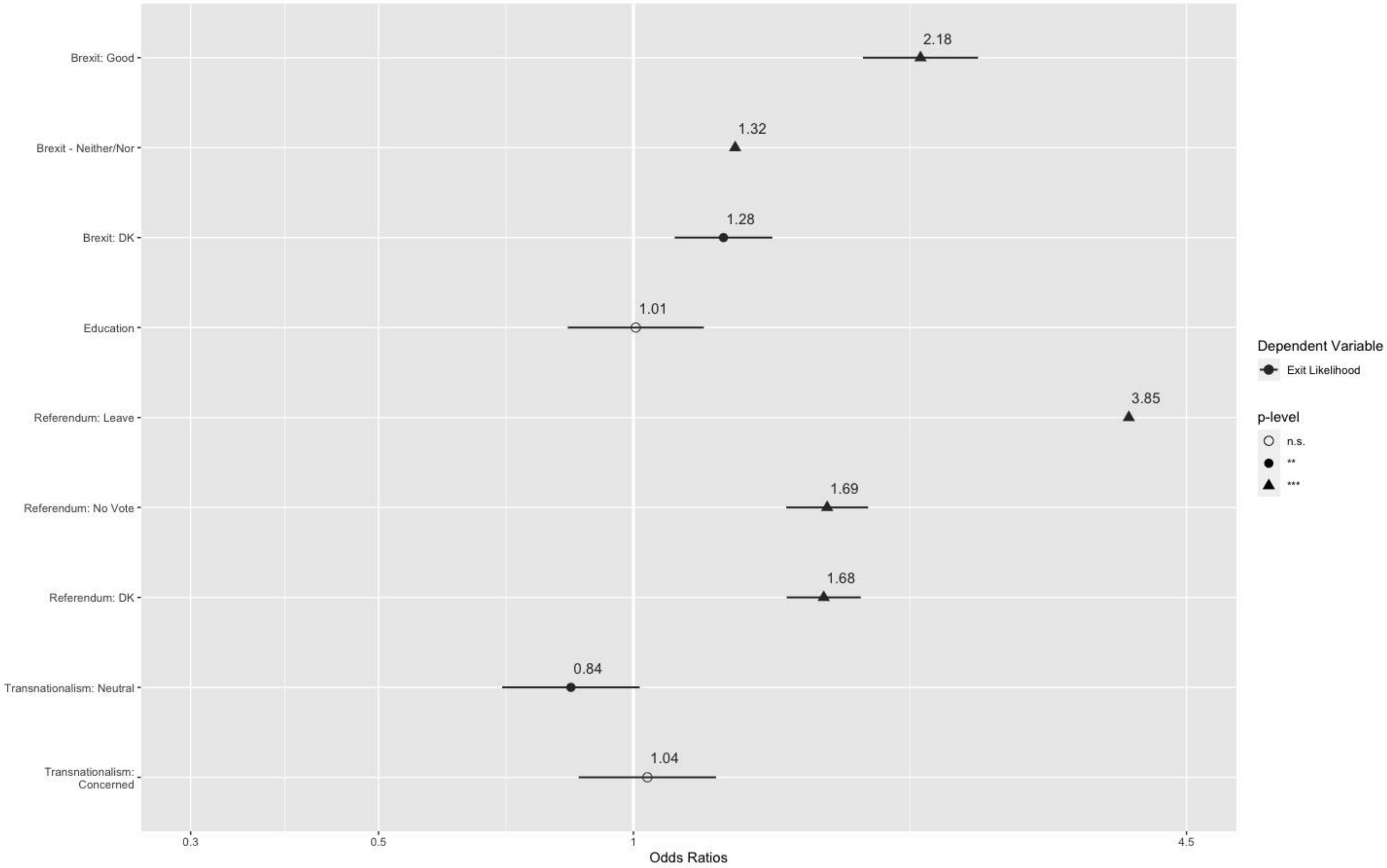

Note: Confidence intervals at 95%

Figure A2: Predicted Probabilities by Country (Model 2)

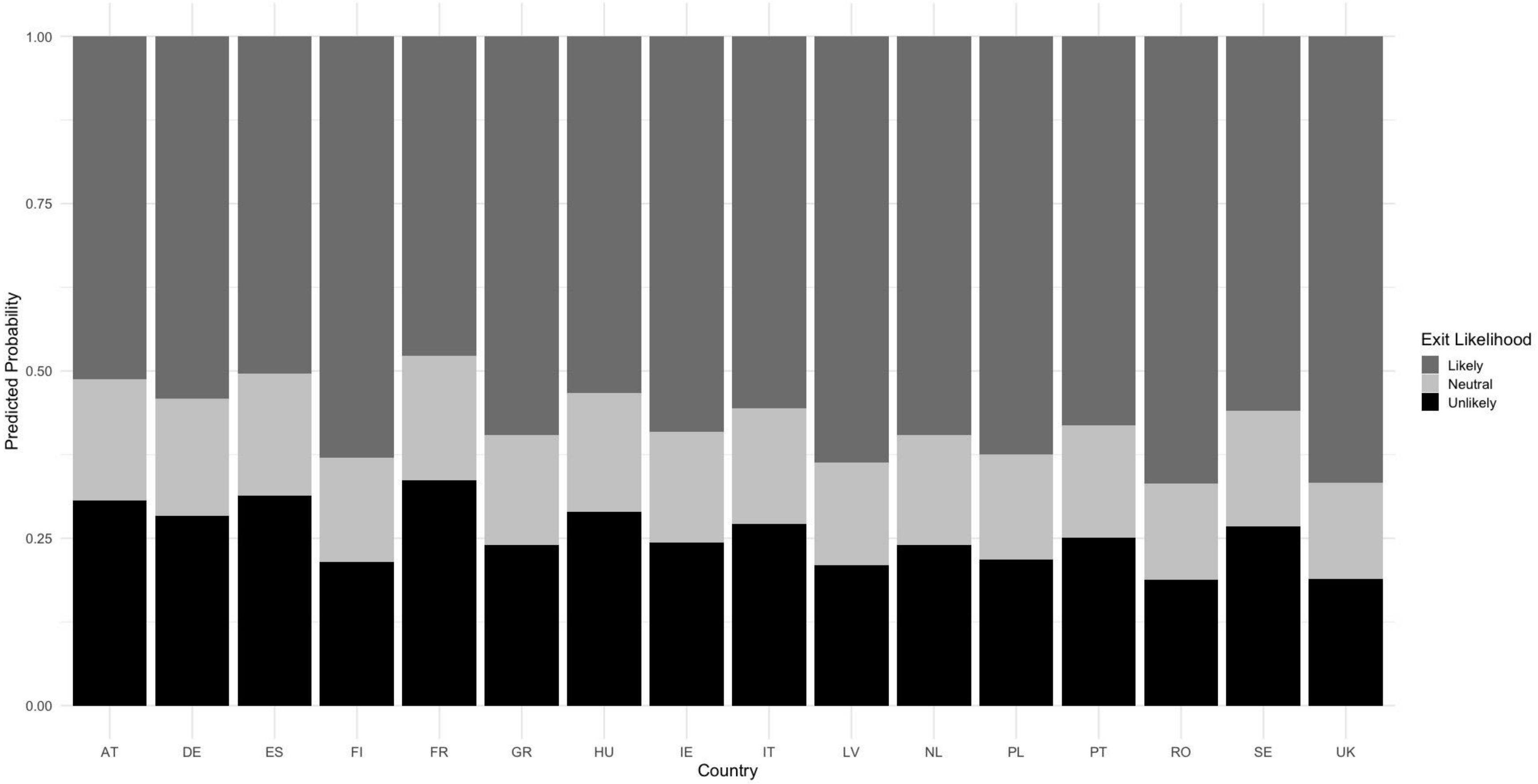

Figure A3: Pearson Correlation Matrix (Model 2)

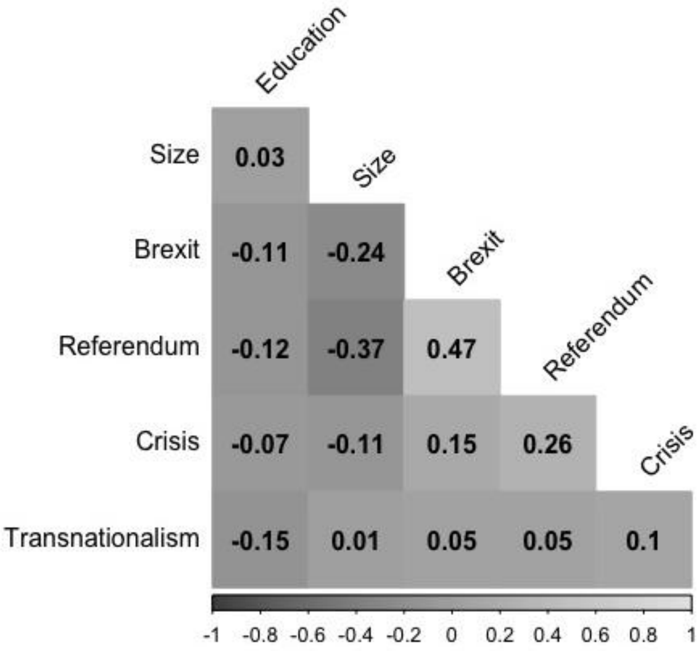

Note: Ordinal categorical data transformed into numeric outputs, with non-scalable ('Don't Know', 'Would Not Vote') responses excluded.
